# HBV Precore G1896A Mutation Promotes Malignancy of Hepatocellular Carcinoma by Activating Endoplasmic Reticulum Stress to Enhance Aerobic Glycolysis

**DOI:** 10.1002/mco2.70365

**Published:** 2025-09-03

**Authors:** Baoxin Zhao, Hongxiu Qiao, Zhiyun Gao, Yan Zhao, Weijie Wang, Yan Cui, Fangxu Li, Yuping Wang, Zhanjun Guo, Xia Chuai, Sandra Chiu

**Affiliations:** ^1^ State Key Laboratory of Virology and Biosafety Wuhan Institute of Virology Center For Biosafety Mega Science Chinese Academy of Sciences Wuhan Hubei China; ^2^ Department of Pathogen Biology Hebei Medical University Shijiazhuang Hebei China; ^3^ Experimental Center for Teaching Hebei Medical University Shijiazhuang Hebei China; ^4^ Department of Gastroenterology and Hepatology The Fourth Hospital of Hebei Medical University Shijiazhuang Hebei China; ^5^ Division of Life Sciences and Medicine University of Science and Technology of China Hefei Anhui China

**Keywords:** aerobic glycolysis, endoplasmic reticulum stress, HBV G1896A mutation, hepatocellular carcinoma

## Abstract

Hepatitis B virus (HBV) precore G1896A mutation is closely associated with poor prognosis of liver disease. We previously revealed that the G1896A mutation could enhance HBV replication and promote hepatocellular carcinoma (HCC) cell growth both in vitro and in vivo. However, the in‐depth mechanisms by which this mutation promotes the malignancy of HCC still need to be explored. Here, we examined the activation of endoplasmic reticulum (ER) stress and glycolysis in HBV G1896A mutation–associated HCC. Bioinformatics, chromatin immunoprecipitation assay and dual‐luciferase assay were performed to give insight into the underlying molecular interaction between ER stress and glycolysis. Here, we observed that HBV G1896A mutation also promoted HCC cell invasion and migration. Furthermore, HBV G1896A mutation induced ER stress, and specifically, PERK‐ATF4 pathway was responsible for the HCC cell malignancy. Mechanistically, PERK‐ATF4 signaling induced transcriptional activation of PFKFB3, a key gene in the process of glycolysis. Finally, in vitro rescue experiments and in vivo efficacy studies revealed that the ATF4‐PFKFB3 axis is necessary for the HCC tumor growth and metastasis. These results highlight that the ER stress and glycolysis are involved in the HCC‐promotion function of HBV G1896A mutation, providing new insights into HBV‐related HCC.

## Graphical abstract

The HBV precore G1896A mutation exacerbates HCC progression by enhancing ER stress and glycolysis. Our study demonstrates this mutation activates the PERK‐ATF4 pathway, which transcriptionally upregulates glycolytic enzyme PFKFB3, driving HCC cell proliferation and metastasis. Through bioinformatics and experimental validation, we established the ATF4‐PFKFB3 axis as a critical mediator of HBV‐related HCC malignancy, offering potential therapeutic targets.

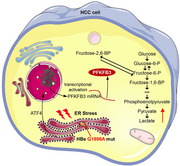

## 1 Introduction

Hepatocellular carcinoma (HCC) is one of the malignant tumors with high incidence and mortality rates worldwide. In spite of great developments in surgery, radiotherapy, and immunotherapy in the past few years, the survival rate for HCC patients is still below 20% [1]. Consequently, the identification of novel tumor biomarkers is critically needed to enhance early diagnosis of HCC and optimize molecular targeted therapeutic approaches, thereby improving patient survival outcomes. As the most common carcinogen for HCC, hepatitis B virus (HBV) is responsible for over 50% of HCC cases worldwide [2]. Chronic HBV infection contributes to the progression of HCC through complicated mechanisms, such as the insertion of viral genome into host cancer genes, the induction of hepatocyte genomic instability, and the activation of cancer promotion signaling pathways [3].

The HBV genome is approximately 3.2 kb and contains four partially overlapping open reading frames (ORFs), including S, C, P, and X, which encode the HBV surface protein (HBs), core protein (HBc), polymerase, and X protein (HBx), respectively [4]. Due to the lack of proofreading ability in the reverse transcriptase enzyme system, a large collection of genetic variants arises in HBV genome [5]. Accumulating evidence from clinical and epidemiological studies indicates that the complex mutational landscape of the HBV genome contributes, via multiple molecular mechanisms, to the development or progression of HBV‐related HCC [6, 7, 8]. The preC/C gene is a hot region where many mutations clustered, and these mutations exhibit strong clinical correlations with the clinical outcome of HCC patients. G1896A is one of the prevalent mutations in HBV precore region, which has been closely related with the development and progression of HBV‐related HCC [9]. We previously found that the HBV G1896A mutation remarkably enhanced HBV replication and promoted the growth of HCC cells. Mechanistically, HBV G1896A mutation activates ERK/MAPK pathway to enhance proliferation and suppress apoptosis in HCC cells [10]. However, the potential mechanism involved in the association between HBV G1896A mutation and HCC prognosis remains to be more precisely explored.

Several initial studies have identified that specific mutations in the HBV gene are connected to the induction of endoplasmic reticulum (ER) stress [11, 12, 13]. The accumulation of misfolded proteins in the ER disrupts ER homeostasis, initiating a cellular condition referred to as ER stress. The unfolded protein response (UPR), including inositol‐requiring enzyme 1 α (IRE1α) pathway, PKR‐like ER kinase (PERK) pathway, and activating transcription factor 6 (ATF6) pathway, is activated in response to ER stress to restore the ER protein processing ability. This restoration is achieved through improving the expression of chaperone proteins, accelerating the degradation of misfolded or unfolded proteins and inhibiting the translation of peptide chains [14, 15]. Sustained ER stress is involved in a variety of dysfunctions in hepatocytes and has been observed in numerous liver diseases, including HCC [16, 17, 18]. Several mutated HBV proteins such as HBs have been found to accumulate in the ER of HBV‐infected hepatocytes to induce ER stress [8, 19, 20], which contributes to the HCC progression, representing a significant mechanism of HBV‐related HCC.

One of the common features of tumor cells is the rewired glucose metabolism, which is characterized by the increased glucose consumption and decomposition of glucose to lactate despite oxygen availability, a phenomenon known as the Warburg effect [21]. The glycolytic intermediates have pivotal roles in synthesizing biomacromolecules, which are indispensable for tumor cell growth [22]. Lots of studies support a role for glycolysis in HCC cell proliferation, in which overexpression or knockdown of key enzymes involved in glycolysis, including hexokinase 2 (HK2), 6‐phosphofructo‐2‐kinase/fructose‐2,6‐biphosphatase 3 (PFKFB3), or lactate dehydrogenase A (LDHA), could induce or reduce the growth of HCC cells [23, 24]. Enhanced glycolysis has been reported to be significantly correlated with the HCC invasion, metastasis, and angiogenesis [25, 26, 27]. The upregulation of glycolysis in HCC is complicated and may result from reprogrammed signaling pathways, which ultimately lead to the upregulation of key glycolytic enzymes. In addition, ER stress plays a crucial role in glucose metabolism and the occurrence and recurrence of HCC [17, 28].

In this study, we aimed to investigate the underlying mechanisms by which HBV G1896A mutation contributes to the progression and aggressive biological behavior of HCC. Our results demonstrated that HBV G1896A mutation promoted HCC cell malignancy via ER stress. Meanwhile, we revealed that HBV G1896A mutation‐induced ER stress could promote glycolysis through the ATF4‐PFKFB3 axis, which contributed to HCC tumor growth, metastasis and exacerbates the prognosis of HCC. Our study provides potential targets that may facilitate the prevention and treatment of HBV‐related HCC.

## 2 Results

### 2.1 HBV G1896A Mutation Promoted HCC Cell Invasion and Migration In Vitro

**FIGURE 1:**
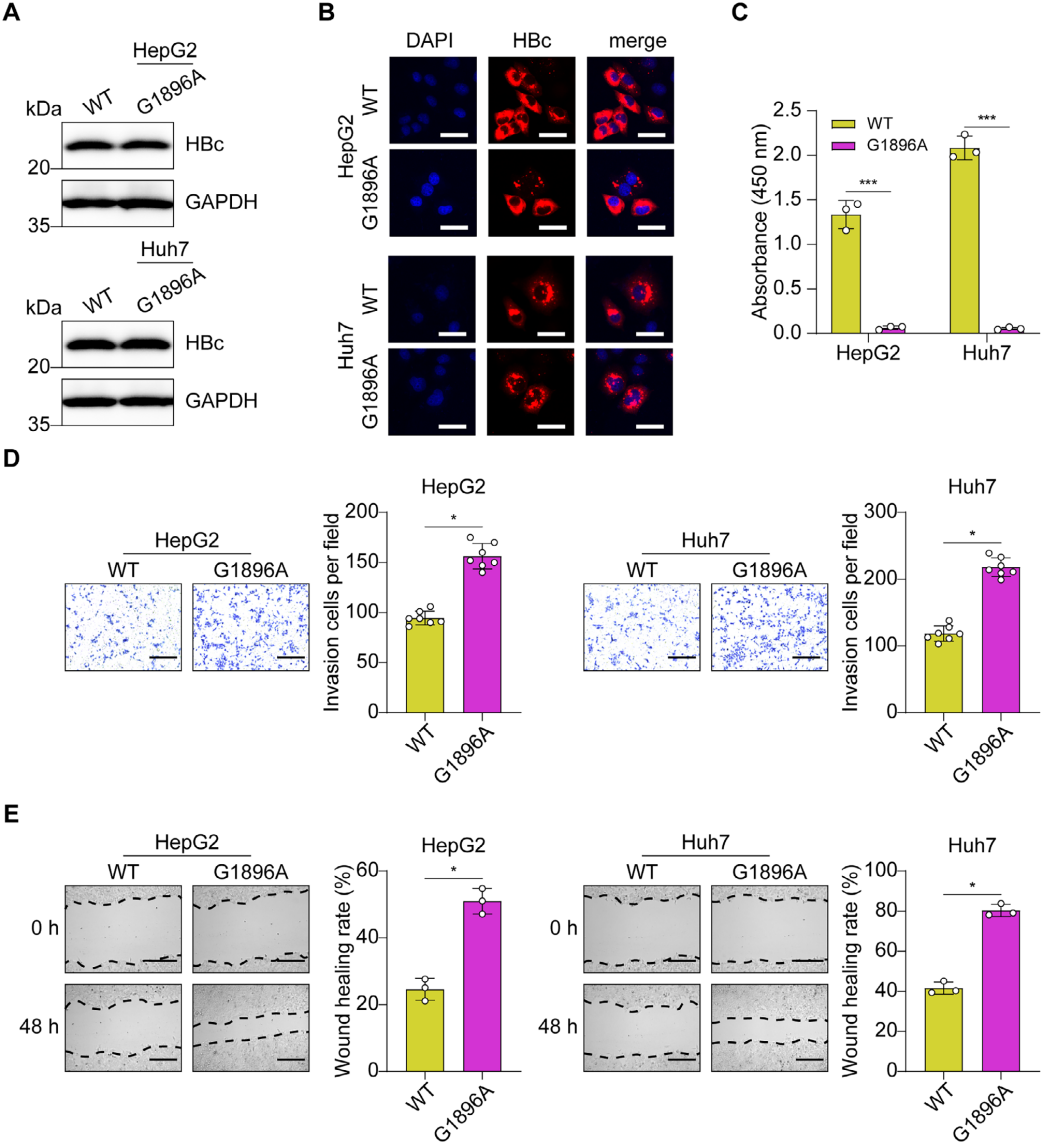
HBV G1896A mutation promoted HCC cell invasion and migration in vitro. (A and B) Western blot analysis and immunofluorescence assay of HBc proteins in HepG2 and Huh7 cells transduced with WT or G1896A HBV preC/C overexpression lentiviral particles. Scale bar, 40 µm. (C) Enzyme‐linked immunosorbent assay of HBeAg in the supernatants of HepG2 and Huh7 cells transduced with WT or G1896A HBV preC/C overexpression lentiviral particles. (D) Transwell assays of the invasion ability in WT or G1896A HepG2 and Huh7 cells. Scale bar, 300 µm. The number of invasion cells was calculated. (E) Scratch wound healing experiments of the migration ability in WT or G1896A HepG2 and Huh7 cells. Scale bar, 300 µm. The wound healing rate of scratches was obtained using ImageJ. All experiments were performed in triplicate, and the results are expressed as the mean ± standard deviation. *p < 0.05, **p < 0.01.

To assess the functional role of HBV G1896A mutation in the HCC tumor metastasis, HepG2 and Huh7 cells were transduced with lentivectors coding for wildtype or G1896A mutant HBV preC/C constructions. As expected, the expression of HBc proteins from the HBV C gene in both wild‐type and G1896A mutant HCC cells was confirmed by immunofluorescence and western blotting analysis (Figure 1A,B). Enzyme‐linked immunosorbent assay (ELISA) results indicated that wild‐type HCC cells, but not G1896A mutant HCC cells, secrete HBeAg into the cell culture supernatants (Figure 1C). Having proven that HBV G1896A mutation promotes the growth of HCC cells [10], we next examined the effects of HBV G1896A mutation on HCC cell invasion and migration. Stable cell clones overexpressing WT or G1896A HBV preC/C genes were obtained as previously described [10]. Transwell and scratch healing assays revealed that HBV G1896A mutation significantly increased the invasion and migration of HepG2 and Huh7 cells (Figure 1D,E).

### 2.2 HBV G1896A Mutation Induced ER Stress

**FIGURE 2:**
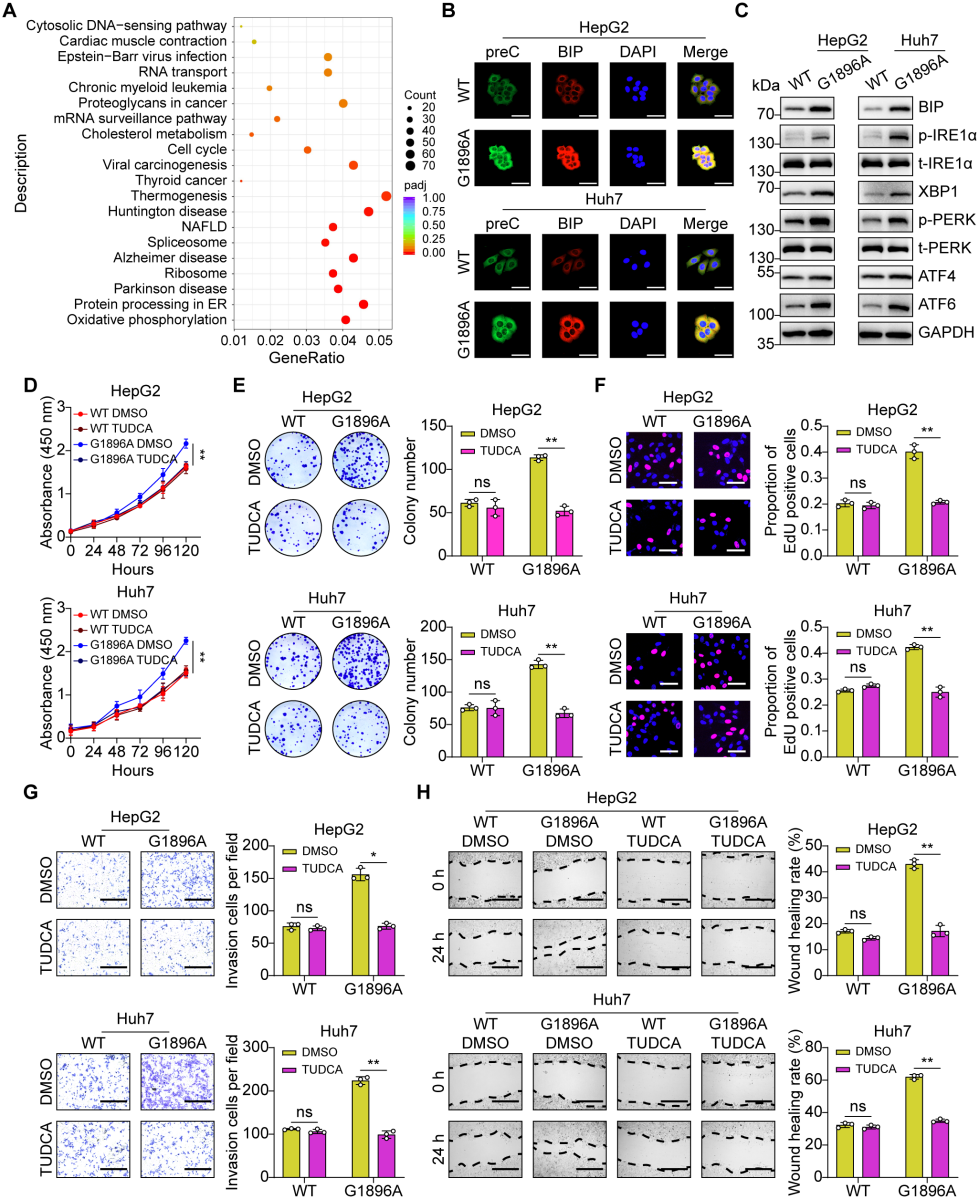
HBV G1896A mutation promoted HCC cell malignancy via endoplasmic reticulum stress. (A) KEGG enrichment analysis of the differentially expressed genes in WT and G1896A HepG2 cells. The size of the bubble represents the number of genes enriched in the indicated pathway, and the color of the bubble represents the probability of the statistical analysis. (B) Immunofluorescence analysis of the expression of WT or mutated preC and BIP in HCC cells. Scale bar, 50 µm. (C) Western blot analysis of ER stress‐related proteins in HepG2 and Huh7 cells. WT or G1896A HCC cells were treated with or without TUDCA (10 µM), and the proliferation, invasion, or migration ability was assayed (D–H). (D) Proliferation of WT and G1896A HCC cells with or without TUDCA‐treated was monitored using CCK8 assay. (E) Colony formation assay of the influence of TUDCA on WT and G1896A HCC cells. (F) EdU incorporation assay was applied to compare the proliferation of HCC cells in different groups. Scale bar, 50 µm. (G) Transwell assays of the invasion ability of WT or G1896A HepG2 and Huh7 cells. Scale bar, 300 µm. The number of invasion cells were calculated. (H) Scratch wound healing experiments of the migration ability in WT or G1896A HepG2 and Huh7 cells. Scale bar, 300 µm. The wound healing rate of scratches was obtained using ImageJ. All experiments were performed in triplicate, and the results were expressed as the mean ± standard deviation. NS: no significance, *p < 0.05, **p < 0.01.

To illustrate the molecular mechanisms underlying the role of HBV G1896A mutation, we performed RNA‐seq in WT and G1896A HepG2 cells. Transcriptome sequencing results showed that 80 genes were upregulated and 223 genes were downregulated in the G1896A HepG2 cells compared with the WT HepG2 cells (|log_2_fold change| ≥ 2, *p* < 0.05). The Kyoto Encyclopedia of Genes and Genomes (KEGG) pathway enrichment analysis on the differentially expressed gene set was applied, and we noted that there was a significant enrichment for upregulated genes in G1896A HepG2 cells, with functions related to the protein processing in ER (Figure 2A), suggesting that the protein processing in ER is enhanced in G1896A HepG2 cells. Considering that the G1896A mutation alters the amino acid sequence of HBeAg precursors encoded by the HBV preC/C gene and may cause protein folding disorders that lead to ER stress, we examined the localization of WT or G1896A mutant HBeAg precursors in ER. The results showed that G1896A mutation caused an increased retention of mutant HBeAg precursors in HCC cells, and an increased expression of binding‐immunoglobulin protein (BIP), a resident chaperone protein localized within the ER lumen (Figure 2B). Notably, we observed that mutant HBeAg precursors colocalized with BIP in the ER. Next, the activation of UPR signaling pathways in WT or G1896A HCC cells was investigated by western blotting. The results showed that G1896A mutation induced the activation of UPR signaling pathway in HCC cells, which was represented by increased protein expression of BIP, X‐box binding protein 1 (XBP1), ATF4 and ATF6, and phosphorylation of PERK and IRE1α (Figure 2C). These results indicated that ER stress can act as a bridge between the HBV G1896A mutation and HCC.

### 2.3 HBV G1896A Mutation Promoted HCC Cell Malignancy via ER Stress

To determine whether ER stress has a functional consequence for G1896A mutation‐related HCC malignancy, we performed loss‐of‐function assays by using tauroursodeoxycholic acid (TUDCA), a hydrophilic bile acid that has chemical chaperone activity, and thus could alleviate ER stress [18]. First, we investigated whether TUDCA could relieve ER stress in G1896A HCC cells. As shown in Figure S1, in G1896A HCC cells, both key indicators of the UPR signaling were decreased upon TUDCA treatment, while these proteins remained unchanged in WT HCC cells. With the alleviation of ER stress, the viability of G1896A HCC cells was significantly inhibited, whereas TUDCA had no effect on WT HCC cell proliferation (Figure 2D). In accordance, through colony formation assay and EdU incorporation assay, we found that there were no remarkable changes in WT HCC cells when treated with TUDCA, while TUDCA treatment significantly reduced the colony number and proportion of EdU‐positive cells in G1896A HCC cells (Figure 2E,F). TUDCA treatment also greatly decreased the invasion and migration ability of G1896A HCC cells, as revealed by transwell and scratch healing assays (Figure 2G,H). These data suggested that ER stress is indispensable in the aggravation of malignancy in HCC cells caused by the G1896A mutation. There are three signaling pathways when the UPR is activated: IRE1‐XBP1, PERK‐ATF4, and ATF6 [15, 29]. Based on these studies, we employed inhibitors specific to each of the three pathways to further identify which signaling pathway plays a key role in the G1896A mutation‐induced malignancy. GSK2606414, a PERK inhibitor, gave rise to a similar suppression on the proliferation, invasion, and migration of G1896A HCC cells as TUDCA treatment (Figures S1 and S2). Nevertheless, the malignant behavior of HCC cells was not influenced by GSK2850163 and Ceapin‐A7, inhibitors of IRE1‐XBP1 and ATF6 pathway, respectively (Figures S1, S3, and S4). Overall, these results suggested that the PERK‐ATF4 signaling pathway is critical for the ER stress‐induced HCC malignancy.

### 2.4 HBV G1896A Mutation Enhanced the Warburg Effect in HCC Cells Through the PERK‐ATF4 Signaling Pathway

**FIGURE 3:**
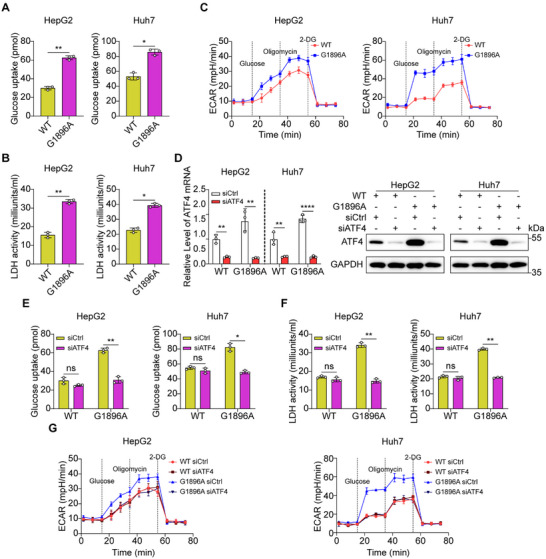
HBV G1896A mutation enhanced the Warburg effect in HCC cells via ATF4. WT or G1896A HepG2 and Huh7 cells were measured for glucose uptake (A) and lactate secretion (B). (C) The extracellular acidification rate (ECAR) of WT or G1896A HCC cells was monitored using the Seahorse assay. (D) Control or ATF4 siRNA was transfected into WT or G1896A HCC cells for 48 h, and the ATF4 expression was detected by qRT‐PCR and western blot analysis. WT or G1896A HCC cells with or without ATF4 knockdown were measured for glucose uptake (E), lactate secretion (F), and glycolysis rate (G). All experiments were performed in triplicate, and the results were expressed as the mean ± standard deviation. NS: no significance, *p < 0.05, **p < 0.01.

Because Warburg effect is responsible for regulating tumor cell proliferation and metastasis [30, 31], we next analyzed the glucose uptake, lactate dehydrogenase (LDH) activity, and the glycolytic function in WT and G1896A HCC cells. The glycolytic phenotype of G1896A HCC cells was presented as increased glucose absorption and LDH activity (Figure 3A,B). Then, we measured the extracellular acidification rate (ECAR) in WT and G1896A HCC cells, and the results showed that G1896A mutation significantly enhanced the glycolytic capacity of HCC cells (Figure 3C). Since G1896A HCC cells exhibited increased ER stress and glycolysis levels, we wondered if there might be relationships between the PERK‐ATF4 signaling pathway and glycolysis. We transfected siRNA targeting ATF4 in both WT and G1896A HCC cells and tested the glucose uptake, LDH activity, and the glycolytic function in both cells (Figure 3D). The results showed that knockdown of ATF4 could significantly inhibit the G1896A HCC cells glucose uptake and LDH activity (Figure 3E,F). Meanwhile, we found that the silencing of ATF4 suppressed the glycolytic capacity of G1896A HCC cells (Figure 3G). These results supported that the PERK‐ATF4 signaling pathway exerted a prominently promotion effect on glycolysis in G1896A HCC cells.

### 2.5 PERK‐ATF4 Signaling Pathway Induced Transcriptional Activation of PFKFB3

**FIGURE 4:**
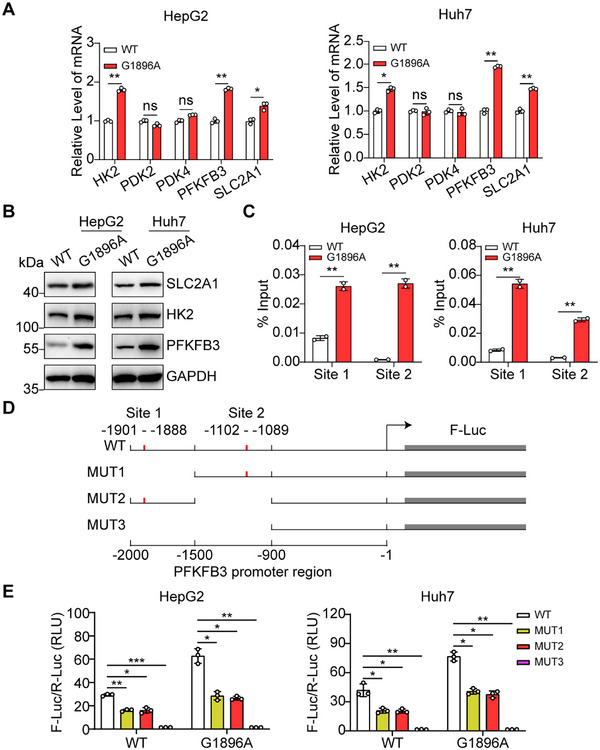
HBV G1896A mutation enhanced the Warburg effect in HCC cells through transcriptional regulation of PFKFB3. (A) Expression of HK2, PDK2, PDK4, PFKFB3, and SLC2A1 in WT or G1896A HCC cells was detected by RT‐qPCR. (B) Western blot analysis of HK2, PFKFB3, and SLC2A1 in WT or G1896A HCC cells. (C) Anti‐ATF4 ChIP assay was conducted in WT or G1896A HCC cells. The PFKFB3 promoter level in the immunoprecipitate was detected by qPCR to determine whether ATF4 could bind to the promoter of PFKFB3. %Input = 100 × 2^(Ct (input)—Ct (IP))^. (D) Potential binding sites of transcription factor ATF4 in the promoter region of PFKFB3 were obtained from JASPAR database (up). Schematic representation of luciferase reporter constructs with WT or truncated PFKFB3 promoter (down). (E) Activity of the luciferase in WT or G1896A HCC cells transfected with indicated luciferase reporter constructs was determined. All experiments were performed in triplicate, and the results were expressed as the mean ± standard deviation. *p < 0.05, **p < 0.01, ***p < 0.001.

According to the above results, we further investigated the expression of key enzymes in glycolysis in G1896A HCC cells. qPCR results revealed an induction on the transcription of solute carrier family 2 member 1 (SLC2A1), HK2, and PFKFB3 genes by G1896A mutation, and western blotting results showed that PFKFB3 was remarkably induced by G1896A mutation in HCC cells (Figure 4A,B). These findings indicated that G1896A mutation increased the glycolysis rate of HCC cells by regulating ER stress and key enzyme in glycolysis. As a transcription factor, ATF4 has been reported to function as a regulator of diverse biological processes [32, 33]. We used the JASPAR database to predict potential ATF4 binding sites in the promoter regions of SLC2A1, HK2, and PFKFB3 (https://jaspar.elixir.no/). The results showed that there were two ATF4 binding sites in the promoter region of the PFKFB3 gene (Figure 4C,D). To test this prediction, we performed ChIP‐qPCR experiments (where ChIP is chromatin immunoprecipitation). As shown in Figure 4C, compared with WT HCC cells, G1896A mutation stimulates the binding of ATF4 and both sites in the PFKFB3 gene promoter region. In addition, we verified the activation of PFKFB3 gene transcription by ATF4 through dual‐luciferase assay. As indicated in Figure 4D, we constructed luciferase reporter system containing WT or potential binding sites‐deficient PFKFB3 promoter. By utilizing this system, we determined that lack of site 1 or site 2 significantly dampened PFKFB3 expression and absence of both sites abolished the expression of PFKFB3 (Figure 4E). These results indicated that ATF4 induced by G1896A mutation was necessary for the PFKFB3 gene expression and glycolysis.

### 2.6 Tumor‐Promoting Function of HBV G1896A Mutation Depends on the ATF4‐PFKFB3 Axis

**FIGURE 5:**
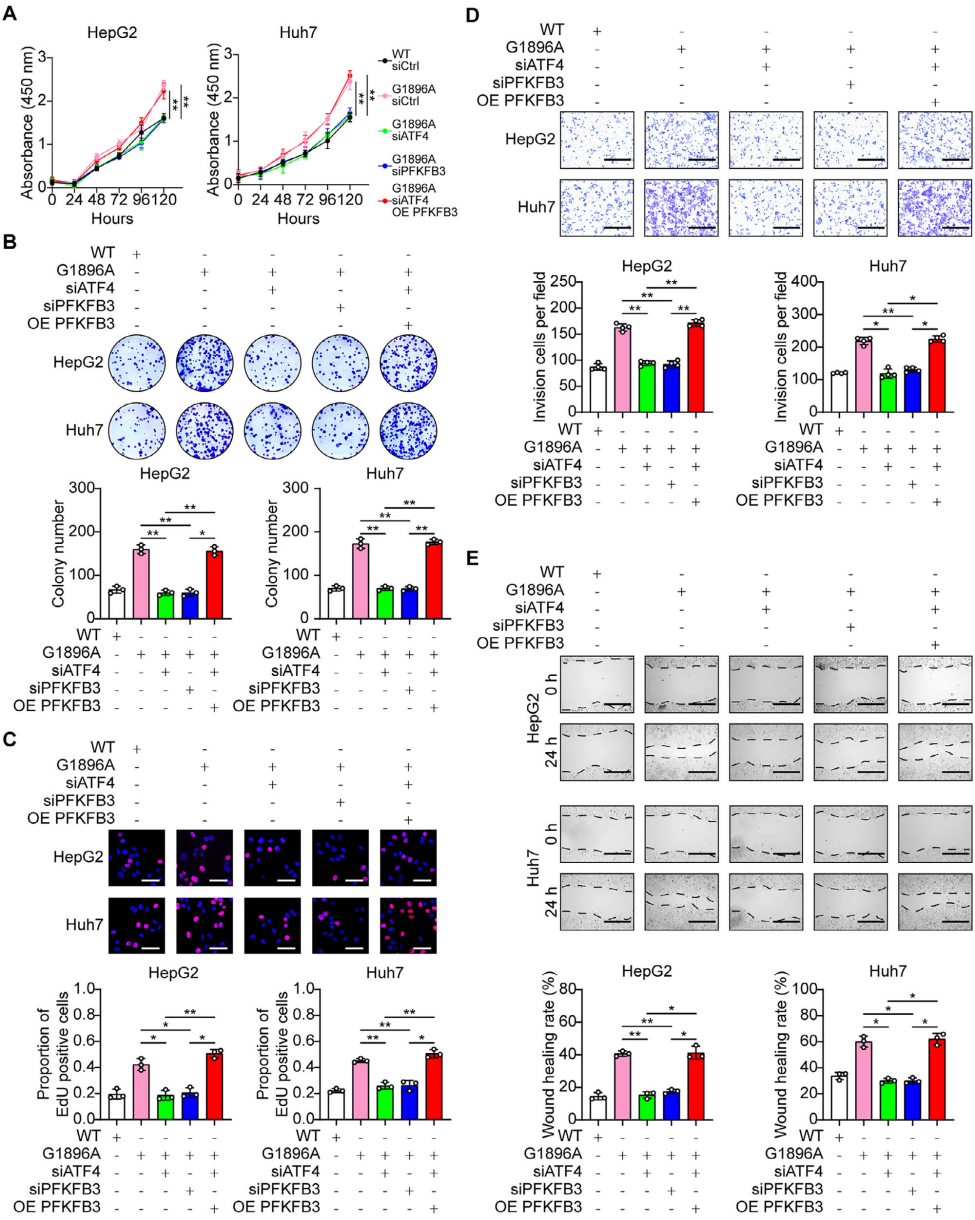
The tumor‐promoting function of HBV G1896A mutation depends on the ATF4‐PFKFB3 axis. WT or G1896A HCC cells were transfected with the indicated siRNA or plasmid, and the proliferation, invasion, or migration ability in vitro was assayed (A–E). The proliferation ability of WT and G1896A HCC cells with or without ATF4 or PFKFB3 knockdown or PFKFB3 overexpression was analyzed by CCK8 assay (A), colony formation assay, (B) and EdU incorporation assay (C). Scale bar, 50 µm. (D) Transwell assays of the invasion ability in the respective groups of WT or G1896A HCC cells. Scale bar, 300 µm. The number of invasion cells was calculated. (E) Scratch wound healing experiments of the migration ability in the respective group of WT or G1896A HCC cells. Scale bar, 300 µm. The wound healing rate of scratches was obtained using ImageJ. All experiments were performed in triplicate, and the results were expressed as the mean ± standard deviation. All experiments were performed in triplicate, and the results were expressed as the mean ± standard deviation. *p < 0.05, **p < 0.01.

**FIGURE 6:**
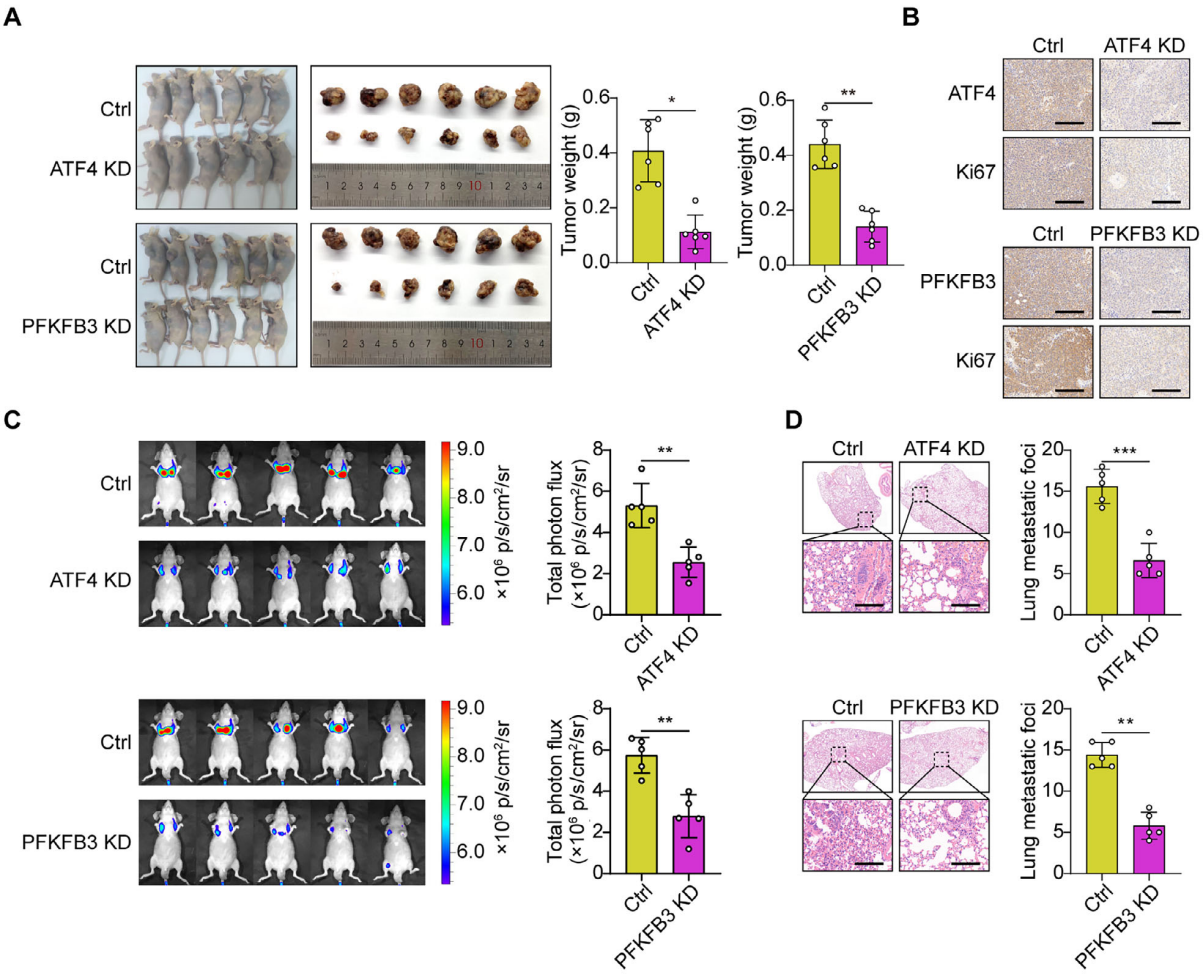
ATF4 and PFKFB3 promote HCC tumor growth and metastasis in vivo. (A) The xenograft nude mice model. Representative tumor xenografts after 25 days of the subcutaneous injection of 1 × 10^6^ indicated G1896A HepG2 cells (left). The final tumor weights after the end of experiment (right). (B) The HCC cell proliferation as determined by immunohistochemical staining of Ki67. (C) Representative images of pulmonary metastasis in mice after tail vein injection with GFP‐labeled HepG2 cells with ATF4 or PFKFB3 stably silenced. (D) Representative images of H&E staining of metastatic foci in the lung. Scale bar, 300 µm. *p < 0.05, **p < 0.01, ***p < 0.001.

Finally, we determined the impact of the ATF4‐PFKFB3 axis on the malignancy phenotype of G1896A HCC cells. We observed that silencing the ATF4 reduced the expression of PFKFB3 (Figure S5). Knockdown of ATF4 and PFKFB3 both would significantly suppress the viability, colony formation ability, and proportion of EdU‐positive cells in G1896A HCC cells, but overexpression of PFKFB3 rescued this proliferation repression (Figure 5A,B,C). Transwell and scratch wound healing experiments were used to test whether the ATF4‐PFKFB3 axis influences the invasion and migration of G1896A HCC cells. ATF4 or PFKFB3 knockdown similarly inhibited the cell invasion and migration, while overexpression of PFKFB3 compensated the depressed invasion and migration ability (Figure 5D,E). Moreover, we explored the role of the ATF4‐PFKFB3 axis in tumorigenesis in vivo. Stable ATF4 and PFKFB3 knockdown and control G1896A HepG2 cells were used to generate subcutaneous HCC xenografts (Figure S6). ATF4 or PFKFB3 knockdown significantly suppressed the xenograft tumor growth, as measured by the end point tumor weight (Figure 6A). As indicated by the immunohistochemistry (IHC) results, ATF4 or PFKFB3 knockdown decreased the staining intensity of Ki67, a proliferation marker of tumor cells (Figure 6B). Furthermore, we evaluated the role of ATF4 and PFKFB3 in HCC pulmonary metastasis. As shown in Figure 6C, the metastatic potential of HCC cells decreased upon ATF4 or PFKFB3 knockdown, and the ATF4 or PFKFB3 stably silenced HCC cells transplanted nude mice exhibited reduced lung metastatic nodules (Figure 6D). These results suggested that the ATF4‐PFKFB3 axis plays a critical role in promoting proliferation and metastasis in the G1896A HCC cells.

## 3 Discussion

Extensive evidence indicate that HBV genome mutations predict poor prognosis in most cases, with multiple signaling pathways involved [9, 34, 35]. In this study, we observed that G1896A‐mutated HBe overexpression generated significant changes in HCC growth and metastasis both in vitro and in vivo. First, in HCC cells, HBV G1896A mutation leads to enhanced ER stress due to the large reserves of the truncated HBe proteins. Along with the ER stress, the UPR responds immediately that is characterized by the activation of three branches of signaling pathways mediated by IRE1α, PERK, and ATF6 transducers. Next, the activation of the PERK‐ATF4 signaling pathway was accountable for the high proliferation and metastasis rates and accelerated aerobic glycolysis in G1896A‐mutated HCC cells. Finally, as a transcription factor, ATF4 binds to the PFKFB3 gene promoter and triggers the transcription.

Metastasis is an important feature of malignant tumors. The dismal prognosis of HCC is relevant to high likelihood of metastasis. After determining that HBV G1896A mutation promotes growth of HCC cells, we examined the effect of G1896A mutation on HCC cell metastasis in the current study. HBV G1896A mutation evidently increased the invasion and migration abilities of HCC cells, implying its role in metastatic progression of HCC. Then, we investigated the molecular pathways that control the metastasis of G1896A HCC cells. By means of transcriptomic analysis, we found that ER stress may be involved in the progression of HCC caused by HBV G1896A mutation. In this regard, the in vitro immunofluorescence colocalization analysis confirmed the ER stress condition in G1896A‐mutated HCC cells. Additionally, the PERK‐ATF4 molecular signaling was proved to be critical in HBV G1896A mutation‐mediated tumor growth and metastasis. Functional relationships of the upregulation of the UPR, especially the PERK‐ATF4 pathway, in the progression of HCC have been investigated in many studies. It was reported that PERK could promote regeneration of intracellular antioxidants and oxidative DNA damage. As a result, it accelerated the tumor cell cycle progression and tumor growth [36]. A previous study on HBx has shown that it induces COX2 expression, an important mediator of hepatic inflammation, through the PERK‐ATF4 pathway [37]. ATF4 has been likewise reported to transcriptionally upregulate the expression of SLC7A11, which drives sorafenib resistance in HCC [32]. Together with our findings, these studies imply the pro‐tumorigenic property of the PERK‐ATF4 signaling pathway in HCC.

**FIGURE 7:**
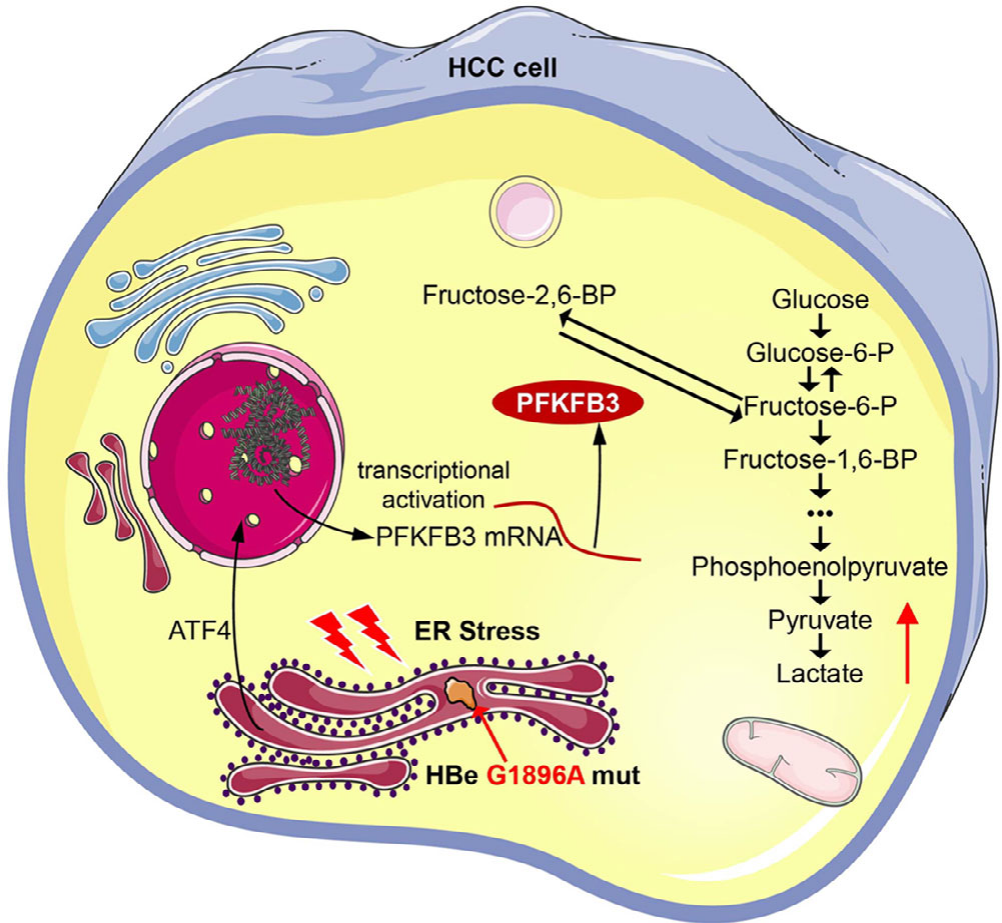
Schematic diagram illustrating the proposed mechanism involved in the HBV G1896A mutation‐induced malignancy of hepatocellular carcinoma.

The reprogramming of glucose metabolism has recently been identified as a hallmark of cancer. Immoderate aerobic glycolysis satisfies the requisites of HCC cell growth and metastasis [38]. Indeed, we found that the aerobic glycolysis was enhanced in G1896A‐mutated HCC cells, which reflected that glycolysis may be responsible for the tumor growth and metastasis in G1896A HCC cells. It has been reported that the acidic tumor microenvironment caused by lactate overproduction allows tumor cells to obtain hypoxia adaptability and metastatic potential [39]. Specifically, we showed that the protein level of PFKFB3 significantly increased in G1896A HCC cells. However, the protein abundances of other key enzymes involved in aerobic glycolysis were only slightly changed. These results indicate a pivotal role of PFKFB3 in the aerobic glycolysis in G1896A HCC cells. To disentangle the regulatory effects of PERK‐ATF4 pathway on aerobic glycolysis, we next adopted loss‐of‐function approaches. When endogenous ATF4 was knocked down, the glycolysis induced by ER stress in G1896A HCC cells was impaired. Hence, we speculated that PFKFB3 might be the hinge that links the PERK‐ATF4 signaling pathway to glycolysis. Using ChIP‐qPCR and dual‐luciferase assays, we defined that ATF4 promoted the expression of PFKFB3 via its transcriptional activation activity. To further interrogate the regulatory effect of ER stress on glycolysis, we proved that PFKFB3 overexpression restored the impaired cell growth and metastatic ability caused by ATF4 knockdown both in vitro and in vivo. Both ER stress and aerobic glycolysis have been shown to be related to adverse outcomes of HCC, and our research found that HBV mutation‐induced ER stress regulates aerobic glycolysis, leading to a poorer prognosis of HCC. Previous reports have also shown that the glucose metabolism reprogramming, in pancreatic cancer cells or colon cancer cells, depended directly or indirectly on the ER stress, suggesting a close relationship between ER stress and aerobic glycolysis in digestive system neoplasms [28, 40]. Nevertheless, our transcriptome sequencing results indicated that oxidative phosphorylation, another aspect of glucose metabolism, was upregulated in G1896A HepG2 cells. The role of oxidative phosphorylation in the malignancy of HBV‐related HCC remains unresolved and warrants additional investigation. Thus, our findings add a new perspective of molecular regulation in HBV‐related HCC progression. In summary, our study demonstrated that HBV G1896A mutation‐induced ER stress regulates the downstream glycolysis gene PFKFB3 through the PERK‐ATF4 pathway to aggravate the malignancy of HCC (Figure 7). It unravels the mechanism by which HBV G1896A mutation regulates host gene expression to maintain a worse prognosis of HBV‐related HCC. In consideration of the high detection rate of HBV G1896A mutation, targeting the key molecules involved in ER stress and glycolysis can be a promising strategy for the treatment of HBV‐related HCC.

## 4 Materials and Methods

### 4.1 Cell Lines and Transfection

The HEK293T cell line and HCC cell lines HepG2 and Huh7 were obtained from the National Collection of Authenticated Cell Cultures (Shanghai, China). All the cell lines were cultured in Dulbecco's modified Eagle medium (Gibco, CA, USA) supplemented with 10% fetal bovine serum under 37°C and 5% CO_2_. The small interfering RNAs (siRNAs) targeting ATF4 and PFKFB3 were synthesized by GenePharma Company (Shanghai, China), and the sequences of the siRNAs used are listed in Table S1. The PFKFB3 overexpression plasmid was constructed by inserting the PFKFB3 coding sequence into the EcoRI site of the pCAGGS vector. Optimal small guide RNAs (sgRNAs) sequences targeting ATF4 and PFKFB3 were identified using the CRISPOR tool (http://crispor.org) and were cloned into the lentiCRISPRv2 plasmid. The construct was transfected into HEK293T cells together with pMD2.G and psPAX2 plasmids to package lentiviruses. Stable expression of exogenous HBV preC/C gene in HepG2 and Huh7 cells was achieved by transduction with HBV preC/C overexpression lentiviral particles as described previously [10]. The recombinant lentiviruses transduced cells were selected with puromycin at 2 µg/mL, and the expression efficiency of HBV preC/C was verified by immunofluorescence microscopy, western blotting analysis, and ELISA. All of the constructed plasmids were sequenced before transfection. According to the instructions, plasmids or siRNAs were transfected into target cells using Lipofectamine 2000 (Invitrogen, CA, USA).

### 4.2 Cell Proliferation Assay

For the cell proliferation test, HCC cells were plated at a density of 2 × 10^3^ cells/well in 96‐well plates and treated with specific ER stress inhibitors. Cell viability was assessed using the CCK‐8 assay (GK10001, GlpBio), wherein 10 µL of CCK‐8 solution was added to each well, followed by absorbance measurement at 450 nm using a microplate reader. For colony formation assay, HCC cells were seeded at a density of 1 × 10^3^ cells/well in 12‐well plates and cultured for 7 days. Following incubation, colonies were stained with crystal violet solution, and the number of colonies was counted. For EdU incorporation assay, HCC cells were seeded in six‐well plates at a density of 1 × 10⁵ cells/well. And 24 h later, cells were incubated with 10 µM EdU (565456, BD Biosciences) for 2 h, followed by fixation with 4% paraformaldehyde and permeabilization with 0.5% Triton X‐100. After reaction with the Alexa Fluor 488‐azide working solution, the cells were stained with DAPI for nuclei visualization. Fluorescent images were acquired using a confocal microscope.

### 4.3 Cell Invasion/Migration Assays

Transwells (354578, Corning) were used for the HCC cell invasion assay. In brief, permeable inserts were coated with Matrigel matrix (354234, Corning), and HCC cells resuspended in serum‐free medium were seeded in the upper chambers (2 × 10^4^ cells/well), and 750 µL of medium containing 10% fetal bovine serum was added to the lower chambers. The plate holding the transwell chambers was incubated in a 37°C incubator for 48 h. Invading cells were stained with crystal violet and counted under a microscope. Scratch wound healing experiments were used to estimate the migration ability of HCC cells. In brief, the cell monolayer was scratched with a pipette tip to generate scratch wounds when cell confluence reached 90%, and the free‐floating cells were rinsed with PBS to remove. At different time points, images of the scrape lines were taken under a microscope, and cell migration rate was calculated using ImageJ software.

### 4.4 Glucose Uptake Assay

Glucose uptake by HCC cells was determined using a Glucose Uptake Assay Kit (ab136955, Abcam) according to the manufacturer's protocol. In brief, HCC cells were treated with different ER stress inhibitors and 2‐deoxyglucose (2‐DG). Then, cell samples were lysed and neutralized. Reaction mix A and mix B were added to sample supernatants and standards successively, and the absorbance at 412 nm was analyzed every 2–3 min on a microplate reader in kinetic mode at 37°C. Finally, the glucose uptake was calculated according to the standard curve.

### 4.5 LDH Activity Assay

LDH activity of HCC cells was determined using a Lactate Dehydrogenase Activity Assay Kit (MAK066, Sigma‐Aldrich) according to the manufacturer's protocol. In short, HCC cells inoculated in 96‐well plates were treated with different ER stress inhibitors and homogenized in 100 µL of cold LDH assay buffer. NADH standards at a series of different dilutions and 50 µL samples were assayed at the same time. The absorbance was measured at 450 nm and the samples were incubated at 37°C for 30 min. Then, the final measurement was taken at 450 nm and the LDH enzyme activity was calculated.

### 4.6 Glycolysis Stress Test

The glycolytic function of HCC cells was measured by the Agilent Seahorse XF Glycolysis Stress Test. The assay was performed with a Seahorse XF Glycolysis Stress Test Kit (103346‐100, Agilent) according to the manufacturer's instructions. Briefly, HCC cells were inoculated in XFp miniplates at 5 × 10^3^ cells/well and treated with different ER stress inhibitors. Then, the medium was changed to Seahorse XF Base Medium, and the cells were incubated in a non‐CO_2_ incubator for 1 h at 37°C prior to the start of the assay. The test consisted of four consecutive stages: basal, glycolysis induction (10 mmol/L glucose), maximal glycolysis induction (1 µmol/L oligomycin), and glycolysis inhibition (50 mmol/L 2‐DG). The data were collected and analyzed using the Seahorse Wave Controller (Agilent).

### 4.7 Dual‐Luciferase Assay

The PFKFB3 promoter with or without different deletions was inserted into the upstream of firefly luciferase in the psiCHECK2 vector. HCC cells were transfected with the luciferase reporter plasmid. After 48 h of transfection, firefly and *Renilla* luciferase activity were measured successively with Dual‐Luciferase Reporter Assay System (E1910, Promega), and *Renilla* luciferase activity was used to normalize the firefly luciferase activity. The induction of the reporters was compared based on the relative luciferase activity.

### 4.8 ChIP Assay

The ChIP assay was performed on HCC cells using a kit (26157, Thermo Scientific) as previously described [41]. The HCC cells were cultured in 10 cm dishes and were treated with 1% formaldehyde to crosslink the chromatin. Resuspend the cells in membrane extraction buffer containing protease and phosphatase inhibitors and vortex to obtain the nuclei. Then, diluted Micrococcal nuclease was added to the acquired nuclei pellets, which were mixed thoroughly to cut the chromatin. When the digestive reaction terminated, the samples were sonicated on ice to break nuclear membrane. The supernatant was transferred to IP buffer, 2 µg of ATF4 antibody was added, and 10% of total samples was used as input. Immunoprecipitation was performed overnight and washed as described. The immunoprecipitated protein‐DNA complexes by the ATF4 antibody were eluted and hydrolyzed by proteinase K to acquire the DNA fragments bound by ATF4. The resulting purified DNA was proceeded to qPCR detection. The sequences of the primers used are listed in Table S1.

### 4.9 Enzyme‐Linked Immunosorbent Assay

The double‐antibody sandwich ELISA technique was used to determine the HBeAg in the HCC cell culture supernatants. Briefly, the supernatants were collected after the lentivirus infection. The HBeAg capture antibody was coated on the surface of the multi‐well plate, and the supernatants samples and controls were added to the wells and incubated at 37°C for 1 h. Subsequently, the plates were blocked for 1 h. The detection antibody conjugated with horseradish peroxidase (HRP) was added and incubated for 1 h. The plate was washed three times with wash buffer, and the absorbance at 450 nm was measured in a microplate reader.

### 4.10 Western Blotting Analysis

Total cellular proteins were extracted from HCC cells using RIPA lysis buffer (89900, Thermo Fisher) supplemented with protease inhibitor and phosphatase inhibitor (K1007, K1015, ApexBio). Protein lysates were separated by SDS‐PAGE and transferred onto PVDF membranes. After blocking with 5% non‐fat milk in TBST for 1 h, membranes were incubated with the indicated primary antibodies with a dilution of 1:1000 at 4°C overnight, followed by incubation with HRP‐conjugated secondary antibodies (ab205718, ab205719, Abcam) with a dilution of 1:5000 for 1 h at room temperature. The following antibodies from Proteintech were used: IRE1 (27528‐1‐AP), PERK (20582‐1‐AP), SLC2A1 (21829‐1‐AP), HK2 (22029‐1‐AP), PFKFB3 (13763‐1‐AP), and GAPDH (10494‐1‐AP). The following antibodies from Cell Signaling Technology were used: BIP (3177), p‐IRE1α (3294), XBP1 (12782), p‐PERK (3192), ATF4 (11815), and ATF6 (65880). The signals were detected using a chemiluminescence substrate (WBKLS0500, Millipore).

### 4.11 Immunofluorescence Assay

HCC cells were plated and cultured in six‐well plates. At 50% confluency, cells were fixed with 4% paraformaldehyde, permeabilized using 0.5% Triton X‐100, and blocked with 10% goat serum. After blocking, cells were incubated overnight with primary antibodies against HBc (ab8638, Abcam), GRP78 (11587‐1‐AP, Proteintech) and FLAG (M185‐3L, Medical & Biological Laboratories) at 4°C, and subsequently incubated with the Alexa Fluor‐conjugated secondary antibody (ab150080, ab150113, Abcam) for 1 h at room temperature. Following immunostaining, cell nuclei were counterstained with DAPI for 15 min at room temperature. The images were captured on a fluorescence microscope.

### 4.12 In Vivo Proliferation and Metastasis Assays

Male BALB/cA‐nu mice (4‐week‐old) were obtained from Beijing HFK Bioscience Co., Ltd., and were maintained in specific pathogen free conditions. G1896A HepG2 cells (1 × 10^6^) with stable ATF4 or PFKFB3 knockdown were injected into the subcutaneous space of the left flank of nude mice. The mice were euthanized when the tumor major axis of reached 15 mm, and the tumors were surgically excised, weighed, and fixed. Immunohistochemical staining was performed on the tumors to determine the expression of Ki67, a cellular marker of tumor proliferation. Specifically, xenograft tumors were fixed, dehydrated, paraffin‐embedded, and prepared into tissue sections. Then, the tumor sections were deparaffinized and hydrated, and the antigen was repaired with sodium citrate solution. Ki67 antibody (27309‐1‐AP, Proteintech), ATF4 antibody (10835‐1‐AP, Proteintech), and PFKFB3 antibody (13763‐1‐AP, Proteintech) were used to detect the indicated protein expression. For the in vivo HCC lung metastasis assays, the indicated GFP‐expressing G1896A HepG2 cells (5 × 10^5^) with stable ATF4 or PFKFB3 knockdown were resuspended in 100 µL PBS and injected into the lateral tail vein of BALB/cA‐nu mice. The mice were monitored for pulmonary metastasis using in vivo imaging system (PerkinElmer) at the indicated time points. All protocols were approved by the Institutional Review Board, Wuhan Institute of Virology, CAS (WIVA45202401).

### 4.13 Statistical Analysis

Statistical analyses were performed using GraphPad Prism 8.0 (GraphPad Software, MA, USA) software. Data are shown as the mean ± standard deviation of mean. Two‐sided Student's *t* test was used to analyze the differences between groups. A *p* value < 0.05 was considered statistically significant.

## Author Contributions

Baoxin Zhao: data curation, formal analysis, investigation, methodology, visualization, writing‐original draft, writing‐review and editing. Hongxiu Qiao: data curation, software, visualization. Zhiyun Gao: investigation, methodology, validation. Yan Zhao: investigation, methodology, validation. Weijie Wang: formal analysis, funding acquisition. Yan Cui: investigation, validation. Fangxu Li: Methodology. Yuping Wang: methodology. Zhanjun Guo: conceptualization, project administration, resources. Xia Chuai: conceptualization, project administration, writing‐review and editing. Sandra Chiu: conceptualization, funding acquisition, supervision, writing‐review and editing. All authors have read and approved the final manuscript.

## Ethics Statement

All the animal research was carried out in accordance with the rules of Basel Declaration and authorized by the Institutional Review Board, Wuhan Institute of Virology, CAS (WIVA45202401; January 11, 2023).

## Conflicts of Interest

All authors declare no conflicts of interest.

## Supporting information




**Table S1**: Primer sequences for qRT‐PCR, ChIP, siRNA, and sgRNA. **Figure S1**: Three unfolded protein response (UPR) signaling pathways are suppressed by different inhibitors respectively. **Figure S2**: PERK‐ATF4 signaling pathway is indispensable for the ER stress induced HCC malignancy. **Figure S3**: IRE1‐XBP1 signaling pathway is not involved in the ER stress induced HCC malignancy. **Figure S4**: ATF6 signaling pathway is not involved in the ER stress induced HCC malignancy. **Figure S5**: Western blot analysis of protein expression of ATF4 and PFKFB3 in HepG2 and Huh7 cells transfected with the indicated siRNA or plasmid. **Figure S6**: Western blot analysis of the protein expression of ATF4 and PFKFB3 in G1896A HepG2 cells infected with the indicated lentivirus.

## Data Availability

The data that support the findings of this study are available from the corresponding author upon reasonable request.
