# Supplementary material for: HBV Precore G1896A Mutation Promotes Malignancy of Hepatocellular Carcinoma by Activating Endoplasmic Reticulum Stress to Enhance Aerobic Glycolysis
Source: MedComm (2020). 2025 Sep 3;6(9):e70365. doi: 10.1002/mco2.70365 (PMC12409075; doi:10.1002/mco2.70365)
Supplement: Supplementary file 1 — Table S1: Primer sequences for qRT‐PCR, ChIP, siRNA, and sgRNA. Figure S1: Three unfolded protein response (UPR) signaling pathways are suppressed by different inhibitors respectively. Figure S2: PERK‐ATF4 signaling pathway is indispensable for the ER stress induced HCC malignancy. Figure S3: IRE1‐XBP1 signaling pathway is not involved in the ER stress induced HCC malignancy. Figure S4: ATF6 signaling pathway is not involved in the ER stress induced HCC malignancy. Figure S5: Western blot analysis of protein expression of ATF4 and PFKFB3 in HepG2 and Huh7 cells transfected with the indicated siRNA or plasmid. Figure S6: Western blot analysis of the protein expression of ATF4 and PFKFB3 in G1896A HepG2 cells infected with the indicated lentivirus. [file MCO2-6-e70365-s001.docx]

**HBV Precore G1896A Mutation Promotes Malignancy of Hepatocellular Carcinoma by Activating** **Endoplasmic Reticulum Stress to Enhance Aerobic Glycolysis**

Baoxin Zhao^1,2#^, Hongxiu Qiao^3#^, Zhiyun Gao^2#^, Yan Zhao^2#^, Weijie Wang^2^, Yan Cui^2^, Fangxu Li^1^, Yuping Wang^1^, Zhanjun Guo^4^*, Xia Chuai^1^*, Sandra Chiu^2,5^*

^1^State Key Laboratory of Virology and Biosafety, Wuhan Institute of Virology, Center for Biosafety Mega Science, Chinese Academy of Sciences, Wuhan, Hubei, China.

^2^Department of Pathogen Biology, Hebei Medical University, Shijiazhuang, Hebei, China.

^3^Experimental Center for Teaching, Hebei Medical University, Shijiazhuang, Hebei, China.

^4^Department of Gastroenterology and Hepatology, The Fourth Hospital of Hebei Medical University, Shijiazhuang, Hebei, China.

^5^Division of Life Sciences and Medicine, University of Science and Technology of China, Hefei, Anhui, China.

^#^These authors contributed equally to this article.

***Correspondence**: Sandra Chiu, Division of Life Sciences and Medicine, University of Science and Technology of China, Hefei, Anhui, China. E-mail: qiux@ustc.edu.cn;

Xia Chuai, Wuhan Institute of Virology, Center for Biosafety Mega Science, Chinese Academy of Sciences, Wuhan, Hubei, China. E-mail: chuaixia@wh.iov.cn;

Zhanjun Guo, Department of Gastroenterology and Hepatology, The Fourth Hospital of Hebei Medical University, Shijiazhuang, Hebei, China. E-mail: zjguo5886@aliyun.com

| **Table S1 Primer sequences for qRT-PCR, ChIP, siRNA, and sgRNA.** | | |
| --- | --- | --- |
| **Name** | **Forward** | **Reverse** |
| **Primer sequences for qRT-PCR** | | |
| ATF4 | 5'-TAAGCCATGGCGTGAGTACC-3' | 5'-GCGCTCGTTAAATCGCTTCC-3' |
| HK2 | 5'-TGTTACTGATTCTCCTGTC-3' | 5'-TGGTTTCATTGCCCAGACTC-3' |
| PDK2 | 5'-TGCCTGTGCGCCTGGCCAAC-3' | 5'-GATGGTGACCAGGGCGTC-3' |
| PDK4 | 5'-GGCATATAGAAAAAAAGGAG-3' | 5'-CAGAAGCACCACAACACTAGG-3' |
| PFKFB3 | 5'-CGTCTGTGAGGAGCTGAC-3' | 5'-ACCAGGTCCTGGTAGGACTCCC-3' |
| SLC2A1 | 5'-GCTACCCTGGATGTCCTATCTG-3' | 5'-GGCAGCTGGACGTGGACC-3' |
|  |  |  |
| **Primer sequences for ChIP-qPCR** | | |
| PFKFB3 promoter ATF4 binding site 1 | 5'-GGTGGCGAGTCTGATACAGG-3' | 5'-AAGGGTGCCCCAAATCCAAA-3' |
| PFKFB3 promoter ATF4 binding site 2 | 5'-CTCAGATGTTGGCCTTTGCG-3' | 5'-TTTCCCGGAGCCTGAAACAG-3' |
|  |  |  |
| **siRNA sequences** | | |
| ATF4 | 5'-GCCUAGGUCUCUUAGAUGACTT-3' | 5'-GUCAUCUAAGAGACCUAGGCTT-3' |
| PFKFB3 | 5'-AGCUGCCUGGACAAAACAUGTT-3' | 5'-CAUGUUUUGUCCAGGCAGCUTT-3' |
|  |  |  |
| **sgRNA sequences** | | |
| ATF4 | 5'-TAGGAGGTCTCTTAGATGATTACC-3' | 5'-AAACGGTAATCATCTAAGAGACCT-3' |
| PFKFB3 | 5'-TAGGCCCACCATGACGATGACGGT-3' | 5'-AAACACCGTCATCGTCATGGTGGG-3' |


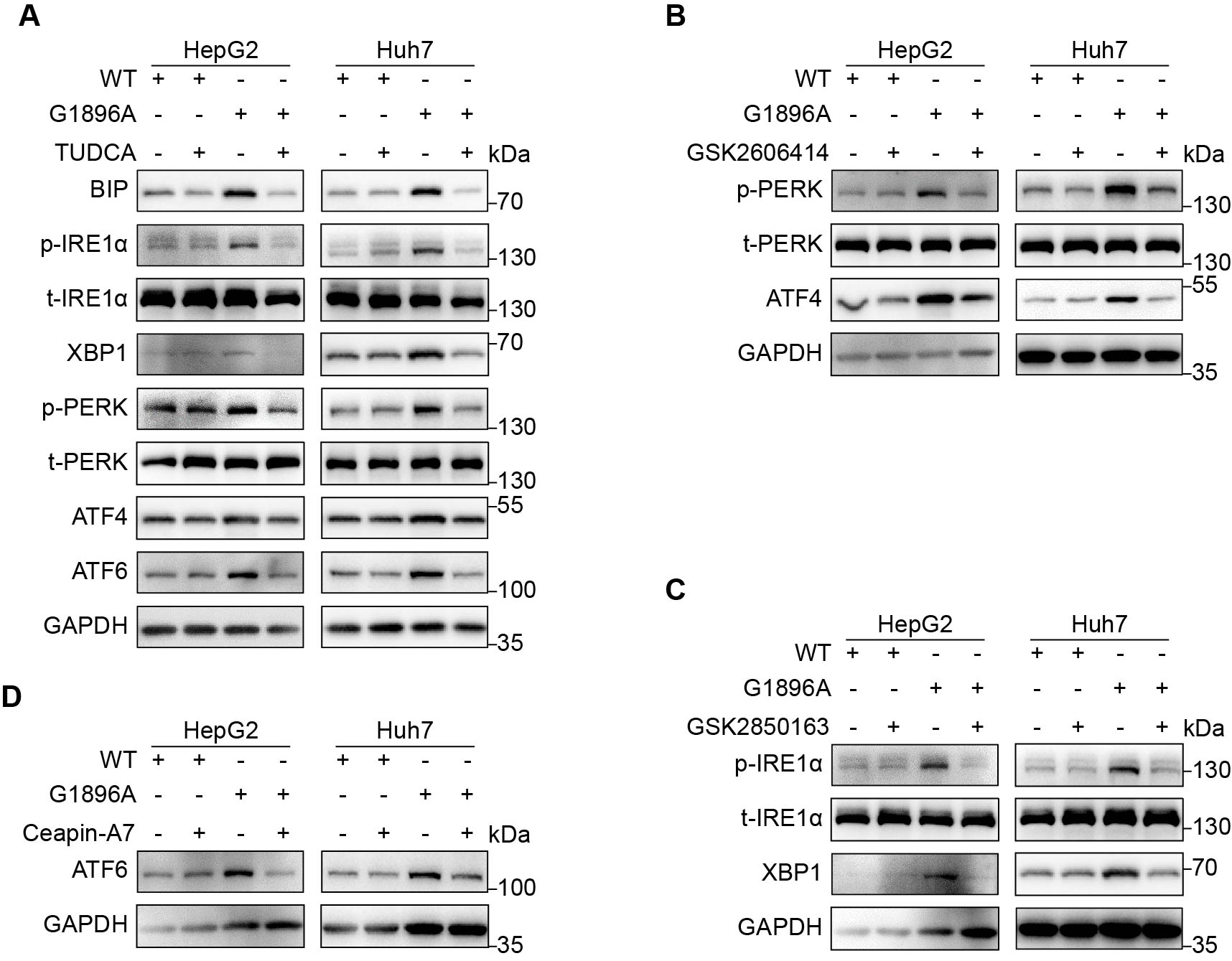


Figure S1 Three unfolded protein response (UPR) signaling pathways are suppressed by different inhibitors respectively. WT or G1896A HCC cells were treated with or without GSK2606414 (1 μM), GSK2850163 (200 nM) or Ceapin-A7 (5 μM) respectively. Proteins involved in three UPR signaling pathways in HepG2 and Huh7 cells were analysed by Western blot.


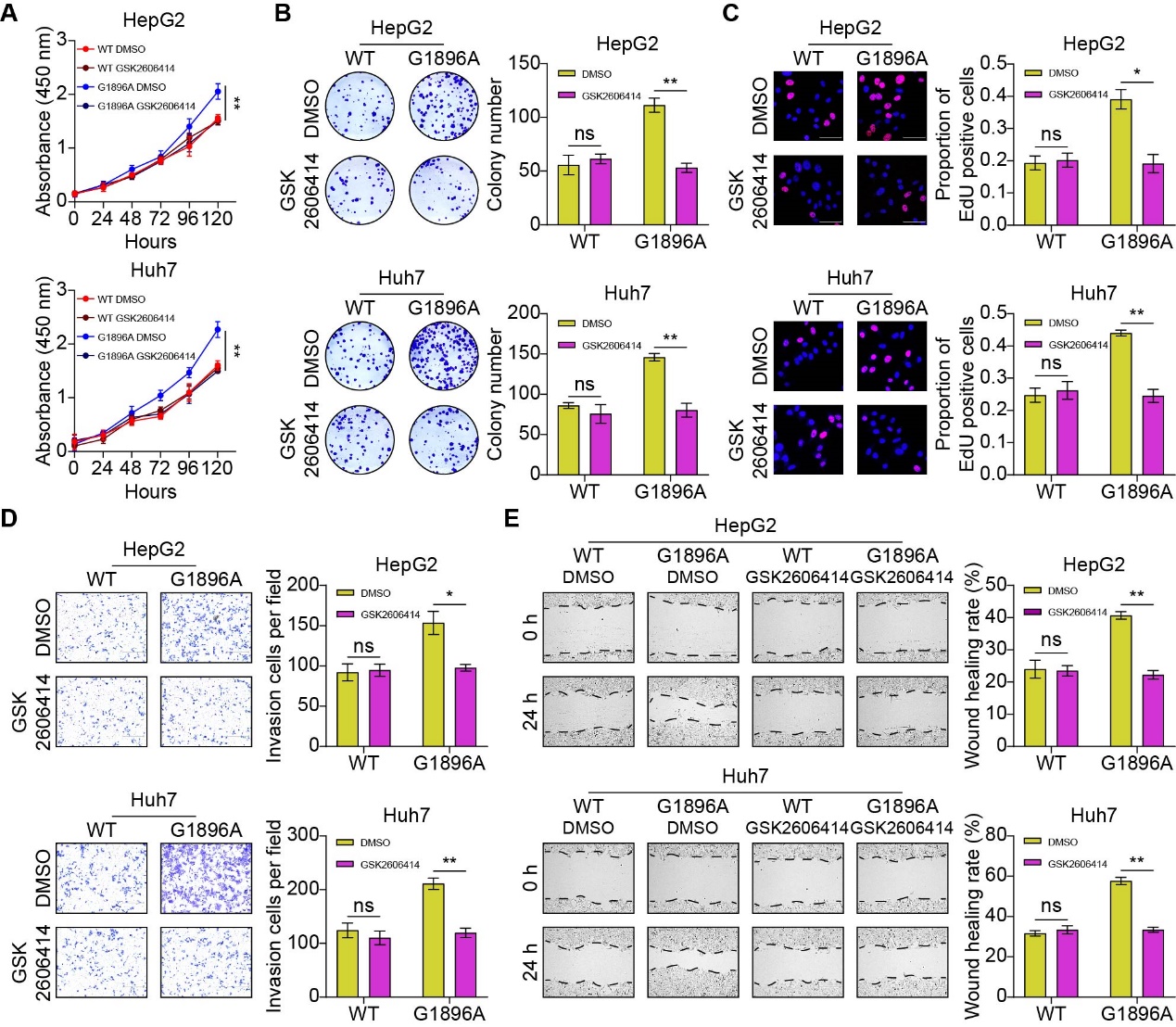


Figure S2 PERK-ATF4 signaling pathway is indispensable for the ER stress induced HCC malignancy. WT or G1896A HCC cells were treated with or without GSK2606414 (1 μM), and the proliferation, invasion or migration ability were assayed (A-E). (A) Proliferation of WT and G1896A HCC cells with or without TUDCA treated were monitored using CCK8 assay. (B) Colony formation assay of the influences of TUDCA on WT and G1896A HCC cells. (C) EdU incorporation assay was applied to compare the proliferation of HCC cells in different groups. Scale bar, 50 μm. (D) Transwell assays of the invasion ability in WT or G1896A HepG2 and Huh7 cells. Scale bar, 300 μm. The number of invasion cells were calculated. (E) Scratch wound healing experiments of the migration ability in WT or G1896A HepG2 and Huh7 cells. Scale bar, 300 μm. The wound healing rate of scratches was obtained using ImageJ. All experiments were performed in triplicate and the results were expressed as the mean ± standard deviation of the mean. NS: no significance, * *P* ＜ 0.05, ** *P* ＜ 0.01.


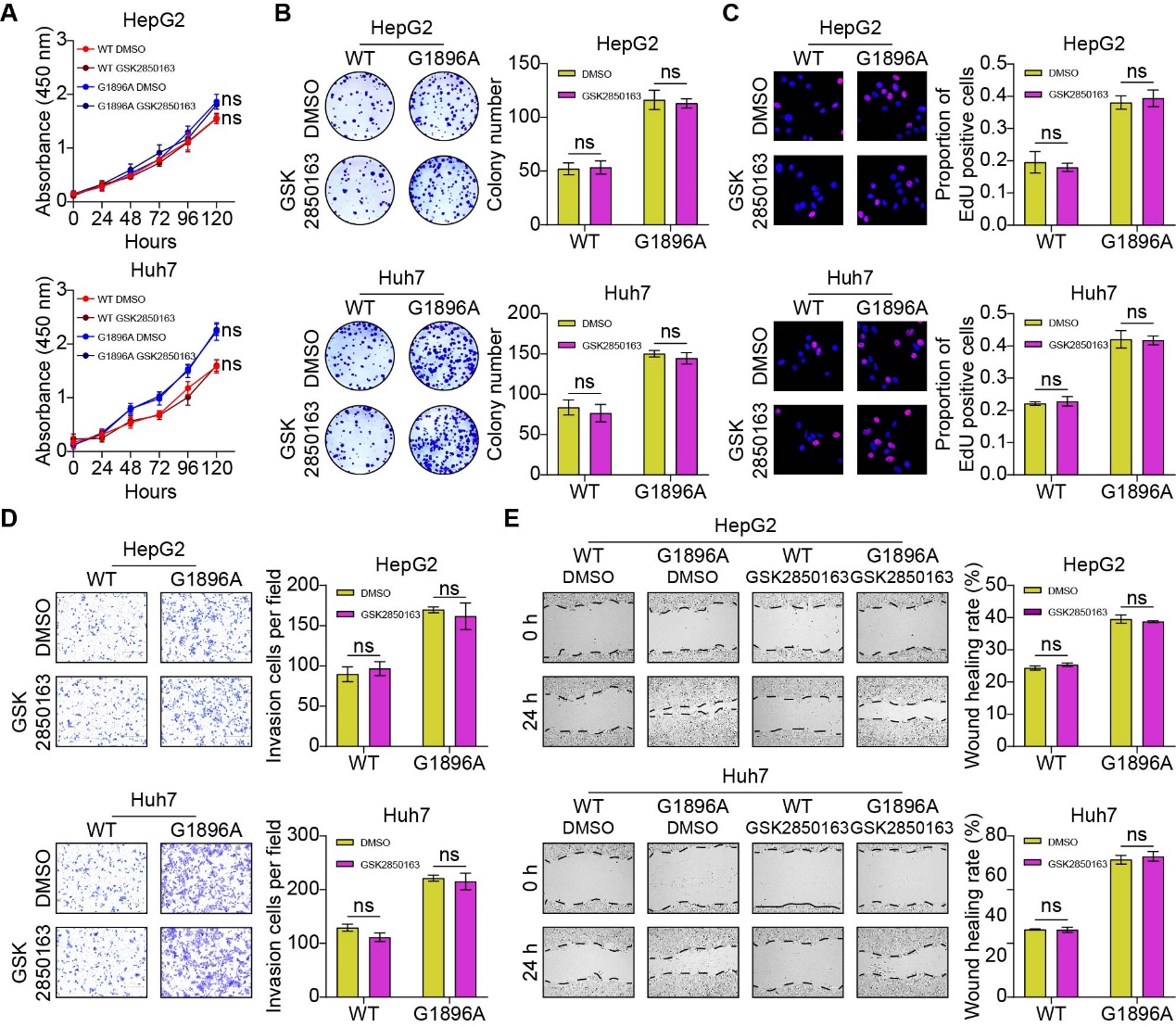


Figure S3 IRE1-XBP1 signaling pathway is not involved in the ER stress induced HCC malignancy. WT or G1896A HCC cells were treated with or without GSK2850163 (200 nM), and the proliferation, invasion or migration ability were assayed (A-E). (A) Proliferation of WT and G1896A HCC cells with or without TUDCA treated were monitored using CCK8 assay. (B) Colony formation assay of the influences of TUDCA on WT and G1896A HCC cells. (C) EdU incorporation assay was applied to compare the proliferation of HCC cells in different groups. Scale bar, 50 μm. (D) Transwell assays of the invasion ability in WT or G1896A HepG2 and Huh7 cells. Scale bar, 300 μm. The number of invasion cells were calculated. (E) Scratch wound healing experiments of the migration ability in WT or G1896A HepG2 and Huh7 cells. Scale bar, 300 μm. The wound healing rate of scratches was obtained using ImageJ. All experiments were performed in triplicate and the results were expressed as the mean ± standard deviation of the mean. NS: no significance.


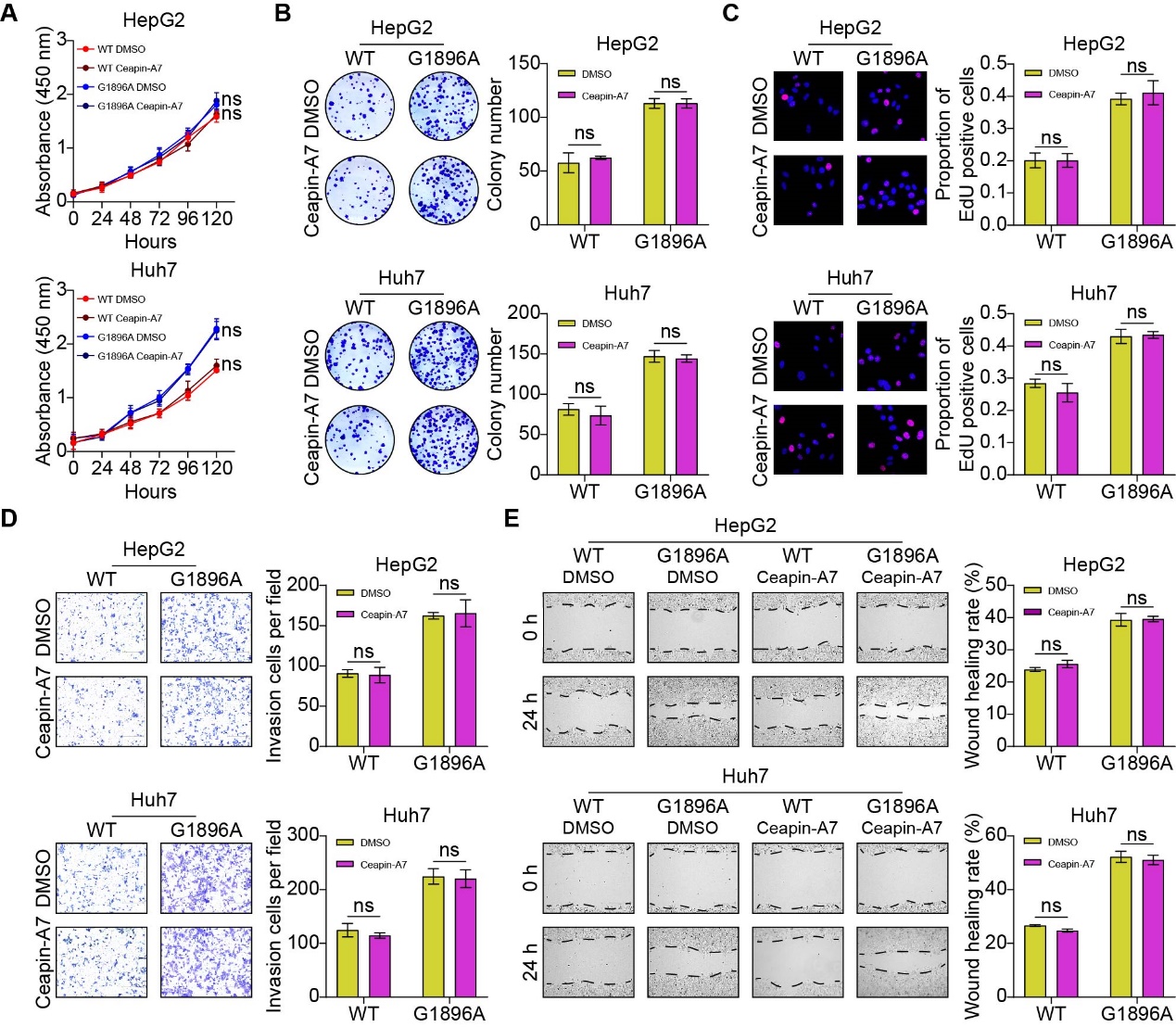


Figure S4 ATF6 signaling pathway is not involved in the ER stress induced HCC malignancy. WT or G1896A HCC cells were treated with or without Ceapin-A7 (5 μM), and the proliferation, invasion or migration ability were assayed (A-E). (A) Proliferation of WT and G1896A HCC cells with or without TUDCA treated were monitored using CCK8 assay. (B) Colony formation assay of the influences of TUDCA on WT and G1896A HCC cells. (C) EdU incorporation assay was applied to compare the proliferation of HCC cells in different groups. Scale bar, 50 μm. (D) Transwell assays of the invasion ability in WT or G1896A HepG2 and Huh7 cells. Scale bar, 300 μm. The number of invasion cells were calculated. (E) Scratch wound healing experiments of the migration ability in WT or G1896A HepG2 and Huh7 cells. Scale bar, 300 μm. The wound healing rate of scratches was obtained using ImageJ. All experiments were performed in triplicate and the results were expressed as the mean ± standard deviation of the mean. NS: no significance.


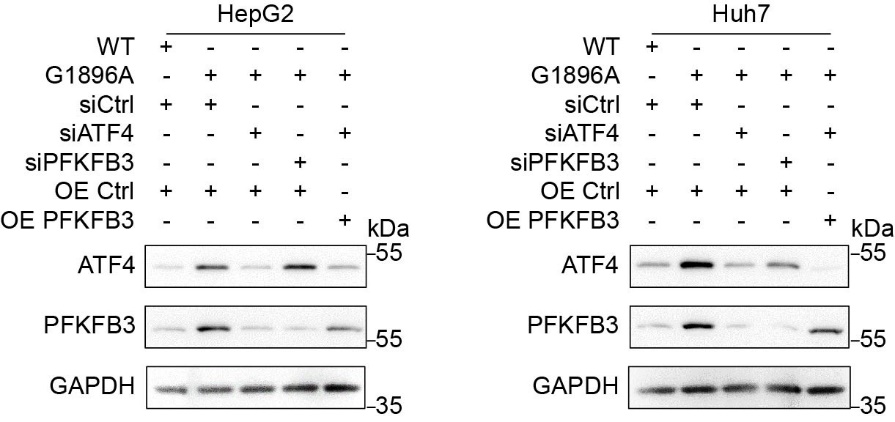


Figure S5 Western blot analysis of protein expression of ATF4 and PFKFB3 in HepG2 and Huh7 cells transfected with the indicated siRNA or plasmid.


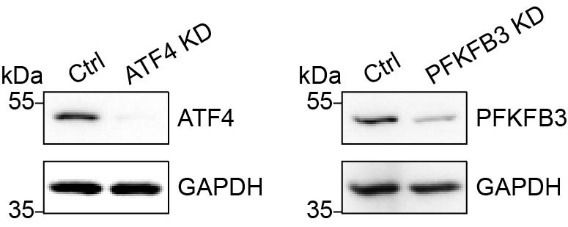


Figure S6 Western blot analysis of the protein expression of ATF4 and PFKFB3 in G1896A HepG2 cells infected with the indicated lentivirus.
